# Increased zinc accumulation in mineralized osteosarcoma tissue measured by confocal synchrotron radiation micro X‐ray fluorescence analysis

**DOI:** 10.1002/xrs.2727

**Published:** 2016-12-21

**Authors:** Mirjam Rauwolf, Bernhard Pemmer, Andreas Roschger, Anna Turyanskaya, Stephan Smolek, Angelika Maderitsch, Peter Hischenhuber, Martin Foelser, Rolf Simon, Susanna Lang, Stephan E. Puchner, Reinhard Windhager, Klaus Klaushofer, Peter Wobrauschek, Jochen G. Hofstaetter, Paul Roschger, Christina Streli

**Affiliations:** ^1^ AtominstitutTU WienViennaAustria; ^2^ 1st Med. Department Hanusch HospitalLudwig Boltzmann Institute of Osteology at the Hanusch Hospital of WGKK and AUVA Trauma Centre MeidlingViennaAustria; ^3^ ANKA synchrotron radiation sourceKarlsruhe Institute of Technology (KIT)Eggenstein‐LeopoldshafenGermany; ^4^ Department of PathologyVienna General Hospital, Medical University of ViennaViennaAustria; ^5^ Department of Orthopaedic SurgeryVienna General Hospital, Medical University of ViennaViennaAustria; ^6^ Orthopaedic Hospital Vienna‐SpeisingViennaAustria

## Abstract

Abnormal tissue levels of certain trace elements such as zinc (Zn) were reported in various types of cancer. Little is known about the role of Zn in osteosarcoma. Using confocal synchrotron radiation micro X‐ray fluorescence analysis, we characterized the spatial distribution of Zn in high‐grade sclerosing osteosarcoma of nine patients (four women/five men; seven knee/one humerus/one femur) following chemotherapy and wide surgical resection. Levels were compared with adjacent normal tissue. Quantitative backscattered electron imaging as well as histological examinations was also performed. On average, the ratio of medians of Zn count rates (normalized to calcium) in mineralized tumor tissue was about six times higher than in normal tissue. There was no difference in Zn levels between tumor fraction areas with a low fraction and a high fraction of mineralized tissue, which were clearly depicted using quantitative backscattered electron imaging. Moreover, we found no correlation between the Zn values and the type of tumor regression according to the Salzer‐Kuntschik grading. The underlying mechanism of Zn accumulation remains unclear. Given the emerging data on the role of trace elements in other types of cancer, our novel results warrant further studies on the role of trace elements in bone cancer. Copyright © 2016 The Authors. *X‐Ray Spectrometry* published by John Wiley & Sons Ltd.

## Introduction

Osteosarcoma is the most common primary malignant bone tumor with a peak incidence in childhood and adolescence frequently occurring at sites of rapid bone growth^1^ with a second smaller incidence peak in the elderly. While the exact cell of origin for this cancer remains to be ill‐defined, osteosarcoma cells produce osteoid and tumor matrix that can mineralize. Due to the use of neoadjuvant chemotherapy, long‐term survival of patients with osteosarcoma has improved from 10% to 20% to nearly 80% within the last 25 years.^2^, ^3^ However, these rates have not improved in the last 15 years.^2^, ^3^ Therefore, it is essential to obtain more insight into the fundamental biology of osteosarcoma that may lead to new treatment.^4^ Trace elements have recently become a field of interest in various physiological as well as disease processes and especially cancer.^5^, ^6^, ^7^ It was found that trace element levels differ between normal and cancerous tissue.^7^, ^8^, ^9^, ^10^ Moreover, novel approaches using trace elements to treat cancer have recently emerged.^11^

Zinc (Zn) is an essential trace element implicated in several biological processes, and various studies reported significant changes in the levels of Zn in different cancer types.^8^, ^9^, ^10^, ^12^ Zn is also an important trace element for bone metabolism.^13^ Zn levels are known to affect the proliferation rate of osteoblasts.^14^ The Zn concentration is higher in bone than in most of the other tissues.^13^ Technical advances in recent times allow the spatial characterization of trace elements in tissues. Confocal synchrotron radiation micro X‐ray fluorescence (SR‐μXRF) having a well‐defined depth information (about 20 µm) has proven to be an effective imaging method for qualitative and semiquantitative analysis of spatial distribution of trace elements in different materials.^15^ We have successfully used this technique to characterize spatial distribution of various trace elements in cartilage and bone samples.^16^, ^17^, ^18^ However, little is known about Zn levels in osteosarcoma. The aims of this study were to investigate the Zn content as well as its spatial distribution in human mineralized osteosarcoma tissue and compare it to adjacent normal bone using both confocal SR‐μXRF and quantitative backscattered electron imaging (qBEI) acquiring the signals only from near surface (about 1‐µm‐depth resolution).

## Materials and methods

### Patients

Nine patients (four women/five men) with a highly malignant G3 osteosarcoma underwent wide resection of the tumor and implantation of a tumor prosthesis following neoadjuvant chemotherapy according to standardized protocols.^19^ Eight patients were between 10 and 18 years old, and one patient was 66 years old (Table 1). Seven samples were from the knee joint, and one was from the proximal femur and one from the proximal humerus. Grades of regression were classified histologically according to Salzer‐Kuntschik *et al*.^20^ Details are summarized in Table 1. The study was approved by the ethics committee of the Medical University of Vienna, Austria, and was performed in accordance with the Helsinki Declaration.

**Table 1:** List of analyzed samples

Patient	*n*	Tissue	Age (years)	Regression grade
P1	5	Prox. tibia	11	4
P2	5	Prox. tibia	12	2
P3	5	Prox. femur	66	2
P4	6	Dist. femur	18	2
P5	4	Dist. femur	18	2
P6	3	Dist. femur	17	2
P7	3	Prox. tibia	14	3
P8	6	Prox. fibula	10	3
P9	4	Prox. humerus	10	3

Prox., proximal; Dist., distal.

*n* is the number of the measured areas; regression grades refer to the histological grade of regression as defined by Salzer‐Kuntschik *et al*.^20^

### Sample preparation

**Figure 1:**
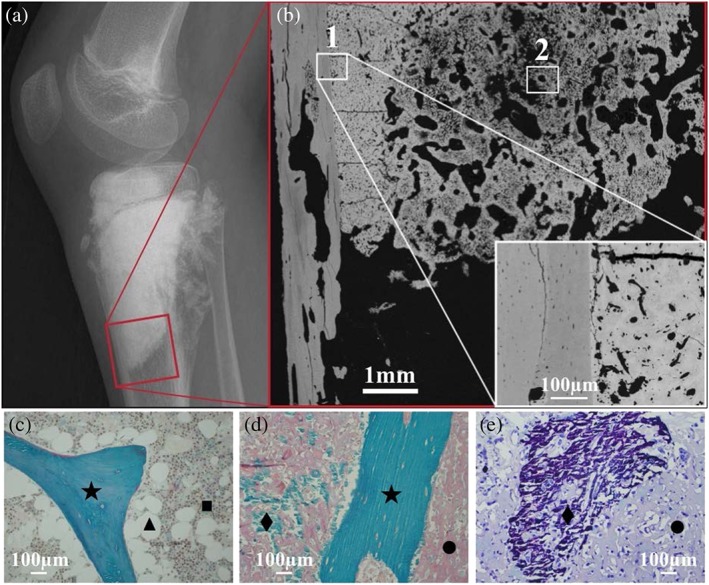
Radiograph (a) of a knee joint with a sclerosing osteosarcoma. The red box indicates the region examined by quantitative backscattered electron imaging. Backscattered electron image (b) showing an overview of a tissue sample. Two regions of interest (ROIs) selected for consecutive Zn synchrotron radiation micro X‐ray fluorescence analysis are indicated. ROI 1 contains healthy bone tissue together with a tumor tissue area, which is mineralized to an extremely high percentage. Further, the tissue matrix itself is mineralized to a much higher degree than normal bone. ROI 2 contains a tumor tissue area, which is mineralized to a much lower percentage than in ROI 1. Representative histological images of tissue sample sections from the same patient: (c) healthy trabecular bone area with intact bone marrow compartment; modified trichrome Goldners staining: green mineralized trabecular bone feature (★), brownish bone marrow cells (■) and white fat cells (▲). (d) Tumor‐affected bone tissue: normal trabecular bone (★), non‐mineralized (•) and mineralized (♦) tumor matrix, (e) region of mineralized (♦) and non‐mineralized (•) tumor matrix (Giemsa staining).

All bone/tumor tissue samples were dehydrated and fixed in a gradient of ethanol concentration (50% to 100%) and embedded in polymethylmethacrylat. The sample blocks were trimmed by a low‐speed diamond saw (Buehler Isomet, Lake Pluff, USA), and 3‐µm‐thick tissue sections were cut by a hard microtome (LEICA SM2500; Leica Microsystems GmbH, Wetzlar, Germany) for histological examinations (Fig. 1). Modified trichrome Goldners and Giemsa staining was performed in representative samples. Consecutively, the blocks were ground with sandpaper with decreasing grit size and finally polished by silk cloths loaded with diamond grains (3 µm and 1 µm) using a precision polishing device (PM5; Logitech Ltd., Glasgow, UK). A flat surface of the sectioned tissue area is crucial for qBEI and SR‐μXRF measurements. The bone tissues in the sample blocks obtained by this procedure were of varying thickness in the 2‐ to 3‐mm range. Afterward, the sample blocks were coated with a thin carbon layer by vacuum evaporation (Agar SEM Carbone coater; Agar Scientific Limited, Essex, UK) to avoid electrical charging effects during backscattered electron imaging. A more detailed description of the sample preparation can be found in previous publications.^21^, ^22^

### Quantitative backscattered electron imaging

Quantitative backscattered electron imaging gives a signal proportional to the average atomic number of the target material. For bone calcium (Ca) – the major bone constituent with the highest atomic number (*Z* = 20) – prevails the signal that allows one to determine the degree of tissue matrix mineralization (Ca content) at each pixel area of the imaged tissue section. In the qBEI images, bright areas describe higher mineralized matrix and dark areas lower mineralized matrix. A digital scanning electron microscope (DSM 962; Zeiss, Oberkochen, Germany) equipped with four quadrant semiconductor backscattered electron detector was used. The scanning electron microscope was operated at 20 keV beam energy, and regions of interest (ROIs) were imaged with 200× nominal magnification (pixel resolution 1 µm). The information depth of the qBEI depends on the mineralization and is in the range of 1–1.5 µm. An example of ROIs, in which consecutively Zn content measurements/mapping were performed, is shown in Fig. 1 as well as histological images of tissue sample sections from the same patient and a radiography of the patient's knee joint. The qBEI measurements for one sample (P1) were previously presented in detail as a case study.^23^ A significantly higher degree of mineralization (Ca content) was reported in the tumor bone tissue compared with the surrounding healthy bone matrix. More information about qBEI can be found elsewhere.^21^, ^22^

### Confocal synchrotron radiation micro X‐ray fluorescence analysis

Confocal SR‐μXRF has proven to be a powerful tool for qualitative and semiquantitative analysis of spatial distribution of trace elements in bone samples.^16^, ^17^, ^18^, ^24^ This method takes advantage of various characteristics of synchrotron radiation such as high photon flux, linear polarization, collimation and the easily tunable primary photon energy that enables to detect absolute amounts in the femtogram range (for medium *Z* elements). Confocal SR‐μXRF uses X‐ray optics (often polycapillary lenses) on the beam side and in front of the detector to define a detection volume from which the fluorescence radiation is detected, which allows to acquire information from the ROI voxel by voxel. Furthermore, the confocal setup eliminates fluorescence radiation (of higher energies in particular of high *Z* elements) from deeper layers and therefore improves the overall spatial resolution. Further details on confocal SR‐μXRF can be found elsewhere.^15^, ^25^

**Figure 2:**
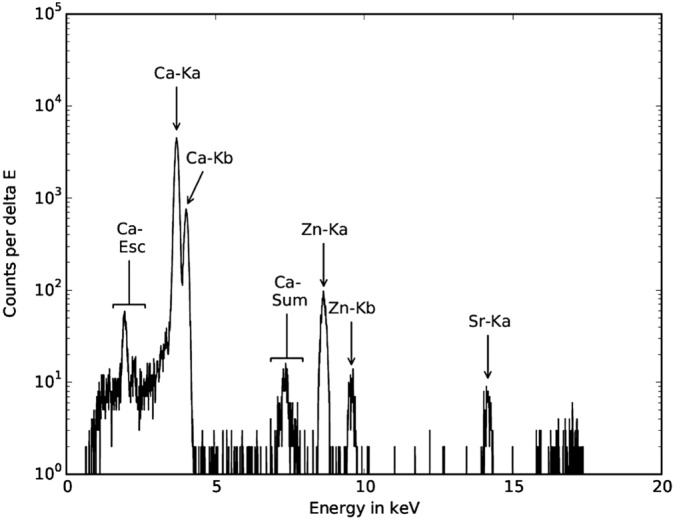
Typical synchrotron radiation micro X‐ray fluorescence spectrum as obtained from a voxel inside a mineralized bone matrix. Data acquisition time was 3 s.

Synchrotron radiation μXRF measurements were performed with a confocal setup at the FLUO beamline at ANKA (KIT, Karlsruhe, Germany)^25^ during multiple beamtimes. For this setup, a W/Si double multilayer was used for monochromatization, keeping the beam exit position constant for various energies. The excitation energy was chosen at 17 keV. As focusing optics, two polycapillary half lenses were used. The fluorescence radiation was detected with a 50‐mm^2^ silicon drift detector (Vortex). The detection volume for the setup was estimated by scanning 0.1‐µm‐thick gold (Au) microstructures. As the size of the detection volume is energy dependent, the step sizes were chosen to be slightly smaller than the detection volume for Au‐L*α* (9.711 keV). The step sizes varied between 10 × 10 µm^2^ and 10 × 17 µm^2^ for the different beamtimes. A depth resolution at 9.71 keV (Au‐L*α*) was about 20 µm, meaning that only signals from this layer were detected. Prior to the area scan of a bone region, depth scans were performed at a few selected points of the ROI. The depth of the highest Ca signal was chosen for the complete area scan. The voxel dimensions achieved for that energy were therefore 10 × 10 × 20 µm^3^, respectively, 10 × 17 × 20 µm^3^. Thus, we measured all elements within a layer only and avoided signals having a larger information depth. The acquisition time of the μXRF signal per voxel was chosen by the signal to noise ratio of test measurements. For most samples, 2 s was sufficient. All spectra were dead time corrected. A typical spectrum obtained in a voxel of a mineralized tissue is shown in Fig. 2.

### Data evaluation

The spectra acquired in each voxel were processed using AXIL software.^26^ Net counts per Ca and Zn were converted to text maps (further referred to as elemental maps).

Custom‐made software was written in python (v.3.4)^27^ for further data processing using the following modules: numpy,^28^ scipy^29^ and matplotlib.^30^ The following evaluation steps were performed:

1. All elemental maps were normalized to 100 mA ring current and counts per second (cps).
2. No Zn was detected in the non‐mineralized tumor tissue as well as bone marrow. Therefore, we focused our analysis solely on the mineralized tissue areas. We differentiated between tumor tissue and adjacent normal bone, which can be easily distinguished based on the backscattered electron images. As there was no Zn, a threshold was introduced in the Ca maps to clearly segment between mineralized tissue areas and soft tissue areas and/or embedding medium. This was performed in an adaptive manner using Eqn (1) for each ROI, because of variations in tissue characteristics and experimental conditions on the synchrotron between the different measurements sessions. A value of 0.5 for *level* (a factor with which the difference between maximum and minimum Ca count rate is multiplied) in Eqn (1) turned out to be a suitable level to evaluate the tissue samples. By this way, partially filled volume effects occurring at the edges between the mineralized and adjacent nonmineralized regions were minimized. Thus, regions around small voids could be excluded from Zn evaluation.

(1) $$T_{\text{Bone}}=Ca_{\min}+\text{level}*\left(Ca_{\max}-Ca_{\min}\right)$$

*T_Bone_* is the threshold value for the mineralized tissue. All voxels with a lower Ca count rate than *T_Bone_* are excluded in the Zn map analysis. *Ca*_min_ represents the minimal Ca count rate of a sample area (ROI). *Ca*_max_ stands for the maximal Ca count rate in the same ROI. The variable *level* can be assigned any value between 0 and 1.

**Figure 3:**
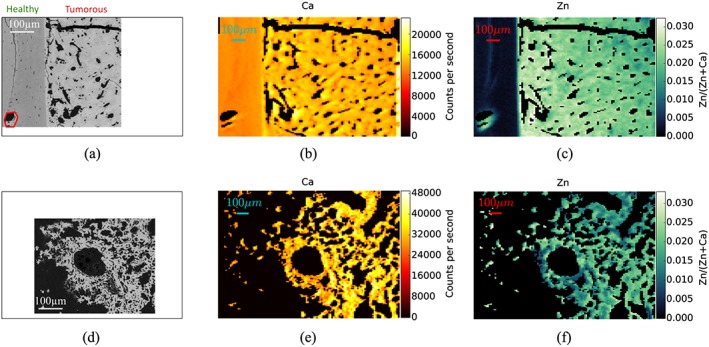
Example of confocal synchrotron radiation micro X‐ray fluorescence (SR‐μXRF) analysis in ROI 1 (a–c) and ROI 2 (d–f) of a mineralized tumor tissue from patient P1: qBEIs are reflecting the mineral content where Ca and Zn mapping was performed (a and d). Ca maps (b and e) are given in units counts per second; Zn maps (c and f) are given as fraction of Zn count rates per total (Ca + Zn) count rates. SR‐μXRF, synchrotron radiation micro X‐ray fluorescence; ROI, region of interest; qBEIs, quantitative backscattered electron imagings.

Areas of all samples were classified as tumorous or healthy by inspecting the corresponding qBEI images. For sample areas containing both healthy and diseased tissue such as shown in Fig. 3(a) to (c), masks were created as binary images in ImageJ (v1.48o, National Institutes of Health, USA),^31^ which then were used by the software to allocate the various pixels of the sample area to healthy bone and to tumor tissue.
Compared with the qBEIs, the elemental maps have a much lower depth resolution. As we only know the surface structure (seen in microscope pictures and qBEI images) of the samples, we corrected count rates for Zn to the Ca signal (Eqn 2). The relative Zn content of a voxel (Zn fraction) was defined as the fraction of the Zn count rate from the total count rates of Ca (Counts*_Ca_*) and Zn (Counts*_Zn_*) together. Otherwise, areas with holes underneath the sample surface might have been evaluated as areas with low Zn content (Counts_Zn_), although related to the available bone tissue [represented by the Ca count rate (Counts_Ca_)], the Zn content might have actually been higher. The calculation of the Zn fraction was chosen over the Zn/Ca‐ratio because it will limit the result to a value between 0 and 1, while the ratio could also result in values bigger than 1.
  (2) $$Zn\;\text{fraction}\;={\text{Counts}}_{Zn}/\left({\text{Counts}}_{Ca}+{\text{Counts}}_{Zn}\right)$$
Mean, standard deviation, 5‐percentile, 25‐percentile, 50‐percentile (= median), 75‐percentile and 95‐percentile of the Zn fraction data were determined for each sample area (ROI) as well as for each sample.
Frequency distributions of voxels with certain Zn fraction values were plotted (Zn histograms).
Boxplots showing the median (black horizontal line), mean (black square), a colored box starting at the 25th percentile and ending at the 75th percentile (containing 50% of the data) and whiskers reaching from the 5th to 95th percentile (and therefore representing a 90% confidence interval) were created.
Moreover, we investigated if the Zn count rate differs with respect to the calcium content in highly mineralized areas *versus* lowly mineralized areas.

### Statistical analysis

Statistical comparison was performed between the groups of median Zn fraction of healthy bone tissue *versus* tumor tissue. Because healthy and tumor tissue was measured from the same sample, a paired test was appropriate. Further, because of the small sample size, it would be unreasonable to assume normal distribution of the measured data; thus, a Wilcoxon signed‐rank test was performed using tabulated critical values.^32^ We correlated the Zn fractions of each tumor to the Salzer‐Kuntschik regression grade in order to see if there is a relationship between the Zn fractions and chemotherapy response.

## Results

As an example, two ROIs within the sample from patient P1 are shown in Fig. 1. ROIs with different tumor characteristics were selected, and resulting Ca count rates and Zn fraction maps with corresponding histograms were determined. The ROI 1 contains both tumor tissue and healthy bone: The classification of the regions is given in the qBEI image (Fig. 3a); one can see that the mineralization and accordingly also the Ca content are distinctly lower in the healthy part than in the tumor area (Fig. 3b). In parallel, the Zn fraction was tremendously increased in tumor area compared with the healthy bone area (Fig. 3c). The qBEI, Ca map and Zn fractions map of ROI 2 (Fig. 3d–f) show an area containing exclusively tumor tissue, which is mineralized to a much lower percentage (meaning that there are less pixels containing mineralized tissue) than ROI 1.

**Figure 4:**
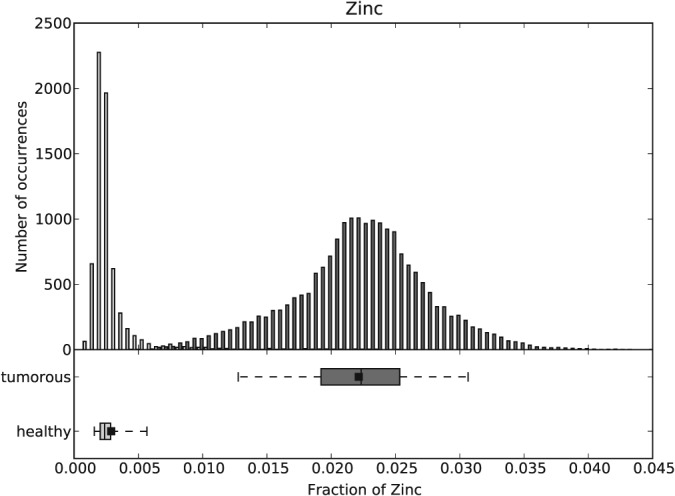
Histogram and boxplots for the Zn fractions over all the healthy (light gray) and tumorous (dark gray) areas of P1.

**Figure 5:**
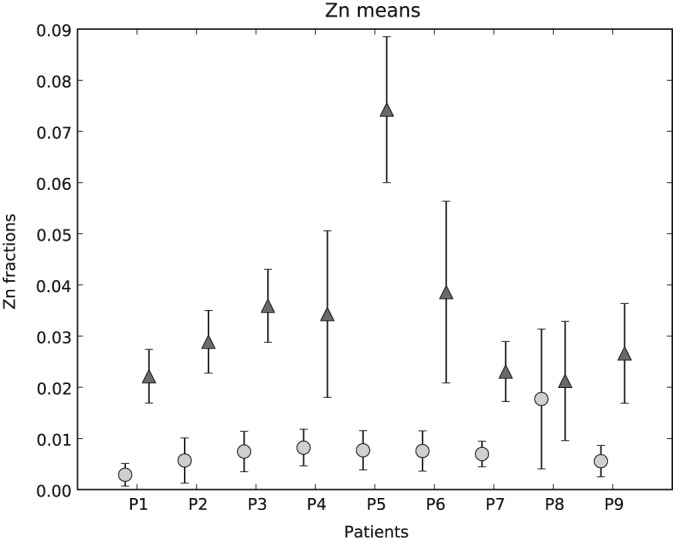
Comparison of means and standard deviations of Zn fractions between healthy bone (light gray circles) and mineralized tumor (dark gray triangles) matrix for each patient.

**Figure 6:**
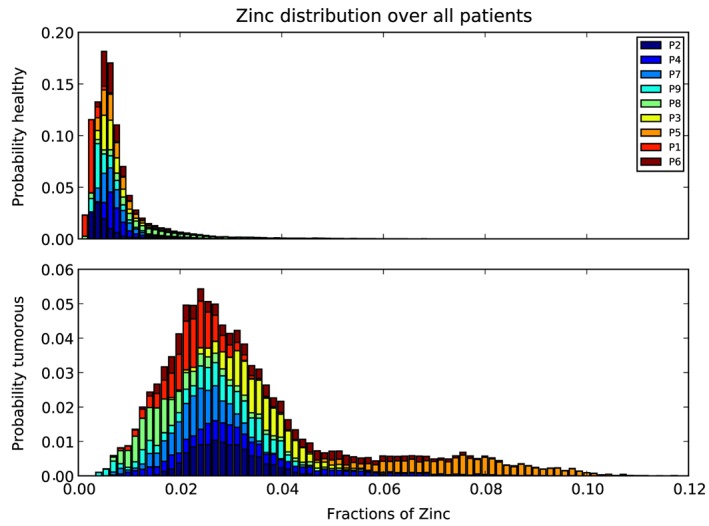
Zn fraction distribution over all patients for healthy (top) and tumorous areas (bottom). The distributions were normalized so that each patient contributes equally to the histogram (independently of the numbers of points measured by patient).

The histogram of Zn fractions of all five ROIs of P1 is shown at once in Fig. 4. Two separated peaks (light gray bars healthy and dark gray bars tumorous region) are visible. Boxplots for the same data set are also shown in Fig. 4 (underneath the histogram). The 90% confidence intervals of the healthy and the tumorous areas are distinctly separated. Only a few outliers of those two distributions overlap. Zn fractions with their means and standard deviations over the healthy and the tumor regions of the nine patients are shown in Fig. 5. As one can see, the Zn mean values for the tumorous regions of a patient are always higher than those for the healthy regions. Figure 6 compares the Zn fraction distribution for healthy and tumorous tissue areas of all patients. The histograms were normalized so that the data of each patient contribute equally and independently of the number of points measured per patient. As one can see, the Zn fraction distribution of the healthy and tumorous areas are clearly separated, and Zn content in the tumorous areas is a lot higher in the osteosarcoma tissue compared with the surrounding healthy tissue.

The Wilcoxon signed‐rank test showed that the median of the tumorous areas of a patient is significantly higher (*p* < 0.005) than the median of the healthy areas of the same patient.

The average $\left(\bar{x}\right)$ of the median ratios (tumorous Zn fractions median divided by healthy Zn fractions median) of all nine patients and its standard deviation $\left(\sigma_{\bar{x}}\right)$ can be reported as 6.05 ± 3.02. While the weighted mean for the nine Zn fraction ratios (mean of tumorous Zn fractions divided by the mean of healthy Zn fractions) and their standard deviations results to $\bar{x}$ ± $\sigma_{\bar{x}}$ = 3.67 ± 0.12. As one can see from both the weighted average and average of the median ratios, the Zn count rate in the tumorous areas of the samples is at least about four times higher than that in the healthy areas of the samples.

Furthermore, we did not find a significant difference in Zn/(Zn + Ca) ratio in lowly mineralized tumor areas *versus* highly mineralized tumor areas, indicating this ratio is relatively unchanged.

Both mean and median of Zn fractions were compared with the histological grade of regression for each patient. No correlation between Zn fraction values and grades of regression were found, indicating that Zn levels did not correlate with the response to chemotherapy.

## Discussion

Synchrotron radiation‐induced confocal μXRF was used to evaluate trace elements in human osteosarcoma tissue. The study revealed tremendously higher Zn fractions in mineralized osteosarcoma regions compared with the healthy bone areas of the same patient. On average, the Zn fraction median for tumorous bone areas was six times higher than that for healthy bone areas. In our samples, we found regions with varying fractions of mineralized tumor tissue areas. Interestingly, we did not find a significant difference in Counts*_Zn_*/(Counts*_Ca_* + Counts*_Zn_*) ratio between tumor areas with a low or high fraction of mineralized tissue, indicating that this ratio is relatively constant.

Other groups have observed changes in Zn levels in connection with various cancer types. For instance, Christudoss *et al.* reported decreased levels of mean tissue Zn (83% and 61%) and plasma Zn (27% and 18%) compared with controls in benign prostatic hyperplasia and prostate carcinoma.^9^ Zn concentration was also found to be significantly decreased in serum for liver cancer compared with in normal serum.^10^ The same study also reported lower Zn tissue concentrations in cancerous and non‐cancerous livers of hepatoma patients compared with those in normal livers. A meta‐analysis of tissue and serum Zn in epithelial malignancies^5^ confirmed the decrease of serum Zn levels in lung, breast, liver, stomach and prostate cancers and Zn tissue levels in prostatic, liver, lung and thyroid cancer. A clear increase of Zn was only found in cancerous breast tissue. Moreover, the results of Al‐Ebraheem *et al.* 12 imply an interrelation between the sites of cancer cell clusters and higher concentrations of Zn, Fe, Cu and Ca in breast tissue.

In our study, we did not see a significant correlation between the Zn fraction values and the response to chemotherapy.

The mechanisms underlying the changes in the Zn concentration between healthy and tumorous mineralized tissue can only be speculated about at this time. As Zn stimulates bone formation,^33^, ^34^ higher Zn fractions in mineralized tumor tissue compared with healthy mineralized tissue seem perspicuous. Zn is an essential trace element implicated in several biological processes including bone metabolism.^13^ Zn ions are used by many proteins to stabilize their structure. For example, Zn finger proteins are among the most abundant proteins in eukaryotic genomes. Many of them are involved in transcriptional regulation.^35^ Moreover, Zn is also a cofactor of several enzymes in bone such as the tissue nonspecific alkaline phosphatase, which plays an important role in bone matrix mineralization.^36^, ^37^

In a recent study, we found homogenous Zn concentration within bone structural units, while increased Zn concentrations were observed in the cement lines separating the differently aged bone structural units.^18^ Interestingly, the Zn concentration was not correlated with the degree of bone matrix mineralization in the different bone structural units, which suggests that Ca is not a significant factor for explaining the Zn concentration in bone and that Zn is therefore likely under homeostatic control.^38^ Zn in the mineralized bone matrix is bond to hydroxyapatite very likely during the early phase of the mineralization process.^39^ It seems that Zn is not just incorporated by ion exchange, but by substitution of vacancy defects of Ca2+.^40^

However, it is noteworthy that no Zn was found in the surrounding soft tumor tissue. As it cannot be entirely ruled out that Zn in soft tissue is lost during sample preparation, cryosections should be analyzed in future studies.

Furthermore, it should be mentioned that our samples were taken after chemotherapy. While it is known that chemotherapy of osteosarcomas has limited effect on the tumor mineralization,^41^ we do not know if the chemotherapy‐induced necrosis has any influence on Zn fractions.

Ambroszkiewicz *et al.* investigated serum bone turnover markers,^42^ such as bone alkaline phosphatase (BALP), which contains Zn as coenzyme,^43^ in children and adolescents with osteosarcoma. Among other things, their findings suggest that BALP levels decrease (from levels similar to healthy patients) during preoperative chemotherapy for all patients and significantly lower values for patients with good prognosis compared with patients with poor prognosis during postoperative chemotherapy and after therapy were found.^42^ Another study^44^ was reported to have found no significantly different serum Zn values in patients with osteosarcoma compared with age‐matched and sex‐matched controls. Future studies combining measurements of Zn distribution in mineralized healthy and tumorous tissue of osteosarcoma patients as well as of BALP values and serum Zn levels at different stages of the treatment could provide new insights into the role of Zn in osteosarcoma.

Due to the complexity of bone structure, there is no acceptable reference material for calibration of the μXRF setup available, which would allow quantifying the absolute trace element concentrations corresponding to the measured count rates. While higher count rates equate higher concentrations, no absolute values (wt%), but only relative values (Zn fractions in the volume), can be given. As the osteosarcoma tissues are not easy to acquire the sample size was rather small (*n* = 9). Heterogeneity of the samples exists concerning age, sex, localization (location of sampling), histological specification and treatment with different types of chemotherapy.

## Conclusion

Synchrotron radiation μXRF allowed us to detect Zn in mineralized osteosarcoma tissue and normal healthy cortical and trabecular bone. Median Zn fractions were found to be a significantly higher in all mineralized osteosarcoma tissue areas compared with normal adjacent bone.

However, the underlying mechanism of Zn accumulation remains unclear. Given the emerging data on other types of cancer and trace elements, future studies will need to take a closer look at the role of trace elements and the clinical outcome of osteosarcoma. Our findings of increased Zn fractions warrant further studies on the role of Zn and bone cancer.
